# Reduction of angiogenesis in chorioallantoic membrane xenografted hepatocellular carcinomas by treatment with a decorin-expressing herpes simplex virus vector

**DOI:** 10.1186/s12985-026-03162-w

**Published:** 2026-04-21

**Authors:** Fanny Frejborg, Oliver Koivisto, Roope Huttunen, Jessica M. Rosenholm, Hongbo Zhang, Hannu Järveläinen, Veijo Hukkanen

**Affiliations:** 1 Pharmaceutical Sciences Laboratory, Faculty of Science and Engineering, Åbo Akademi University, Turku, Finland; 2 Institute of Biomedicine, University of Turku, Turku, Finland; 3 Department of Internal Medicine, Satasairaala Central Hospital, The Wellbeing Services County of Satakunta, Pori, Finland

**Keywords:** Decorin, Herpes simplex vector, Gene therapy, Antiangiogenic agent

## Abstract

Decorin is a proteoglycan that suppresses tumor growth and angiogenesis. We studied whether our recently constructed decorin-expressing herpes simplex virus (HSV) vector can reduce angiogenesis in xenografted liver carcinoma cells in the chorioallantoic membrane model. The vascularized tumors were treated with an overlay dose of our vector. The results show that the treatment reduced angiogenesis in the tumors by 60% (*p* = 0.005) four days after treatment, suggesting that decorin-expressing HSV vectors are a promising strategy for novel cancer therapies.

## Introduction

Hepatic cancer is the third leading cause of cancer related mortality, being often preceded by liver cirrhosis [1, 2]. Decorin (DCN), an extracellular proteoglycan, is pivotal in preventing cirrhosis during healing of the wounded liver [2, 3]. DCN is often found ablated from liver cancers, and its deficiency promotes tumorigenesis of the liver [3–5]. In previous studies, DCN has not only been shown to prevent cirrhosis, but also to reduce angiogenesis and tumor growth [6, 7]. Thus, inducing DCN expression in the liver could be supportive in the treatment of hepatocellular carcinoma.

We previously constructed a DCN-expressing oncolytic herpes simplex virus (HSV) vector with a *γ*_*1*_*34.5* virulence gene deletion, and demonstrated its improved oncolytic potential in vitro in A549 lung adenocarcinoma cells [8]. HSV vectors with a *γ*_*1*_*34.5* deletion are selectively replicative in tumor cells and are safe and efficient clinically, as the HSV vector T-Vec is approved for the treatment of melanoma [9]. We set out to evaluate our novel DCN-expressing HSV vector for anti-angiogenic activity, based on the pivotal role of DCN in angiogenesis [10]. Chicken chorioallantoic membrane (CAM) models are commonly used to study angiogenesis. Huh7 cells were chosen as a model for hepatocellular carcinoma, but also due to their intact interferon pathway [11]. This pathway inhibits the oncolytic activity of the virus itself, allowing to study the effect of DCN on tumor angiogenesis without the interference of the HSV vector.

In this study, we evaluated whether a single dose of topical treatment with our novel DCN-expressing HSV vector could reduce angiogenesis in hepatic xenografted Huh7 cell tumors using a CAM model.

## Materials and methods

### Cells and viruses

Vero cells (CCL-81, ATCC) were maintained in Dulbecco’s modified Eagle medium (Thermo Fisher Scientific), 7% fetal bovine serum (FBS), 5mM GlutaMAX (Thermo Fisher Scientific) and gentamycin. Huh7 cells were maintained in Dulbecco’s modified Eagle medium supplemented with 7% FBS, 5 mM GlutaMAX and gentamycin [12]. The HSV vectors H2252 and H2254 are both *γ*_*1*_*34.5* virulence gene deletion vectors with luciferase transgenes. H2252 has a *DCN* and a blue fluorescent protein transgene. H2254 has a blue fluorescent protein transgene. The viruses were stored frozen in 9% milk, and administered as dilutions in phosphate-buffered saline (PBS).

### Western blotting

Western blotting was performed as previously described [8]. Huh7 cells were infected with 0.1 multiplicity of infection and collected 48 h post-infection. Primary antibodies used were for DCN (Abcam, Cambridge, UK) or the HSV glycoprotein D (Santa Cruz Biotechnology, Texas, US), and for GAPDH (Invitrogen, Massachusetts, US) diluted 1:1000.

### Chicken chorioallantoic membrane model

Fertilized eggs were incubated at 37 °C, 50% humidity, and with rotation every 30 min. On day 3, two ml of albumin was removed with a syringe, the shell windowed, and the eggs were maintained in horizontal position thereafter. On day 7, a ring seal from a cryovial cap was placed on the CAM and the membrane was lightly tapped with a glass rod before adding half a million Huh7 cells suspended in 40 µl PBS and 20 µl growth-factor reduced Matrigel (Corning, New York, US) per egg.

On day 10, the formation of tumors was assessed. 34 eggs in total had formed tumors, and were blindly divided into three groups. All groups were then randomly given a treatment of 14 µl of either PBS, 10^7^ plaque forming units (pfu) of H2252 diluted in PBS or 10^7^ pfu of H2254 diluted in PBS. The treatment was overlaid on the tumor inside the cryovial seal. On day 14, the eggs were sacrificed by placing them on ice. The egg shells were then opened, and the tumors harvested into PBS. The tumors were imaged and halved. The number of free end branches, hallmark of tumor angiogenesis, of the formed vessels in the tumors was calculated to determine the degree of tumor angiogenesis [13]. Half of the tumor was incubated in 4% paraformaldehyde for two weeks and the other half was suspended in medium and homogenized for virus titration, using 5 mm metal beads (Qiagen, Germany) for 10 min using TissueLyser LT (Qiagen).

### Histology

The immunohistochemistry was purchased from the HistoCore service unit at the University of Turku, Finland. A primary antibody against the HSV major nucleocapsid protein VP5 (SeraCare, Massachusetts, US) was used as an indicator of replicative virus.

### Viral titration

The homogenized tumor was titered onto Vero cells as previously described [8]. The sample was diluted 1:2 and then as tenfold dilutions. After 1.5 h of infection, the supernatant was overlaid with DMEM supplemented with 7% FBS, gentamycin and 40 mg/ml immunoglobulin G (HyQvia, Takeda, Tokyo, Japan) [14]. After 3 days, the cells were fixed and stained and quantified for viral plaques.

### q-RT-PCR

DCN mRNA copy numbers were quantified from RNA extracted from homogenized tumors according to previously described methods [8].

### Statistical analysis

The statistical significance was calculated using Mann-Whitney’s non-parametric U test with SPSS statistics 26.0. (IBM, New York, US). The graphs were made using Origin (OriginLab Corporation, Massachusetts, US).

## Results

**Fig. 1:**
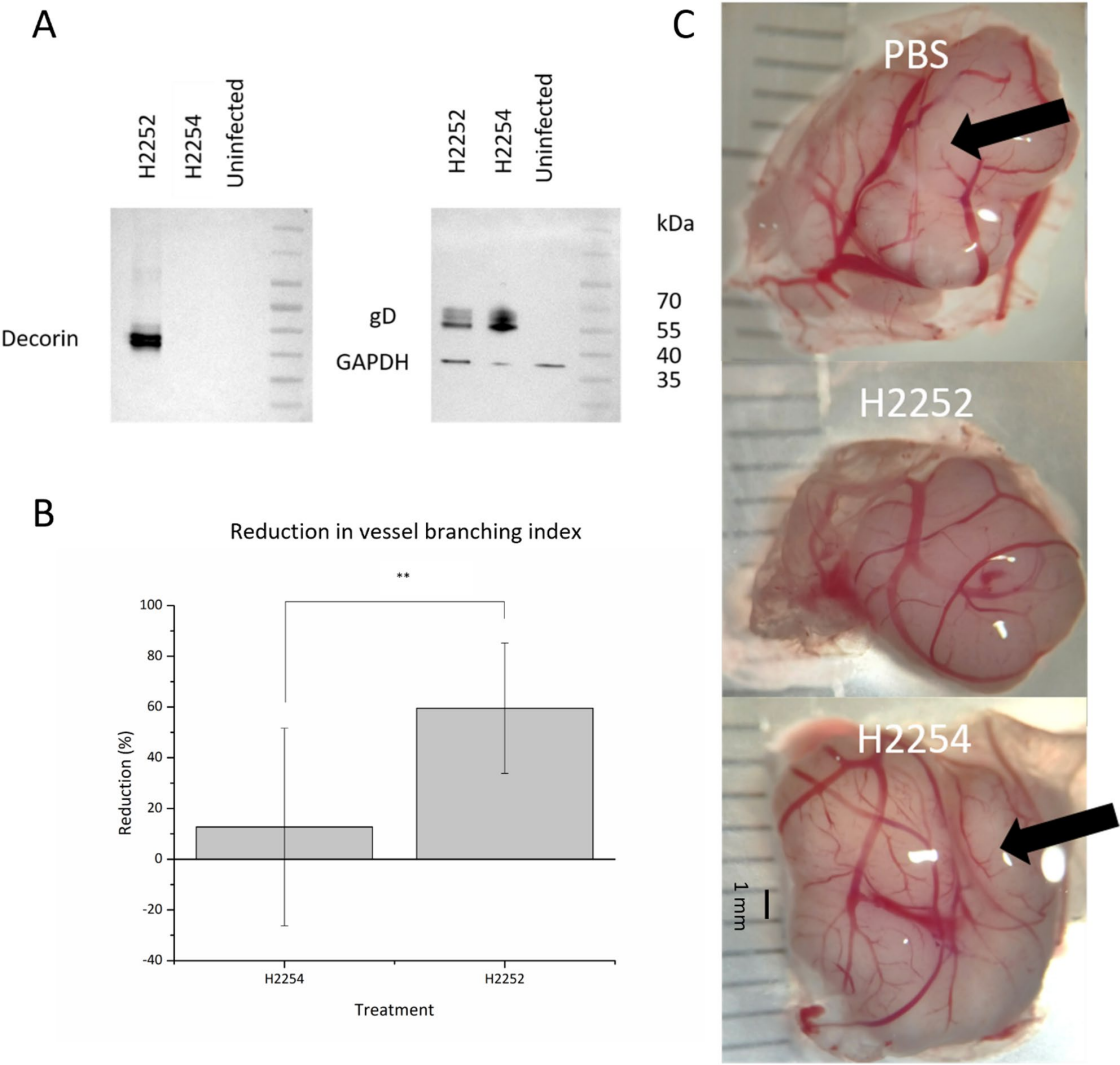
**A** Western blot of Huh7 cells 48 h post vector treatment. Both images show the same blot, with decorin (DCN) on the left panel and GAPDH and viral glycoprotein D (gD) on the right panel. The lane H2252 represents cells infected with DCN-expressing HSV and H2254 its respective control virus. **B** Quantification of vessel branching of Huh7 cell tumors xenografts 4 days post-treatment. The groups were compared to PBS-treated control. Data points = 34. The number of free end branches in the vasculature was counted, ranging from 8 to 75. Asterisk represents p value of 0.005. **C** Representative images of treated Huh7 tumor xenografts 4 days post-treatment, the black arrows depicting examples of free end branches. The scale bar in (**C**) represents 1 mm

Before initiating the study utilizing the CAM model, the ability of Huh7 cells to produce DCN, when treated with H2252, was investigated with immunoblotting. Upon vector treatment of the cells, it was noted that neither HSV vector induced cytopathic effect on the cells. Instead, the gene expression was confirmed using the marker genes of the vectors. The immunoblotting results showed that Huh7 cells have no detectable native DCN production, but when treated with H2252, they produced DCN (Fig. 1A).

Huh7 cells were inoculated onto the CAM of chick embryos. On day 10, embryos had formed visible tumors. The treatment was performed as a single dose overlaid onto the tumors. When the eggs were sacrificed, a total of 34 tumors were harvested.

**Fig. 2:**
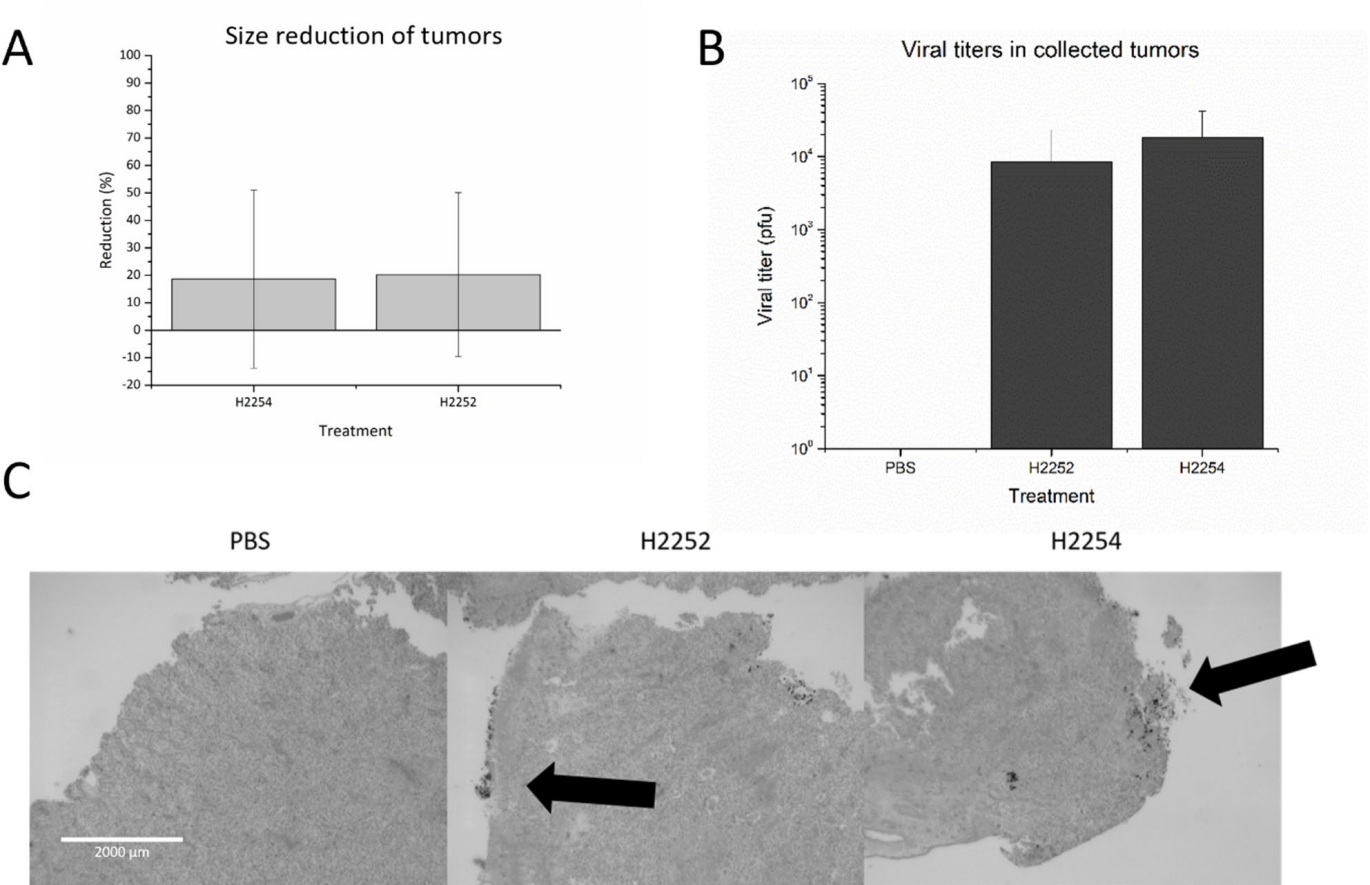
**A** Size reduction of Huh7 tumor xenografts compared to control (PBS) treated 4 days after the treatment. H2252 is the DCN-expressing vector and H2254 is its respective control vector. Data points = 34. The capped bars represent standard deviation. **B** Viral titer in homogenized tumors 4 days post treatment. **C** Immunohistological staining of viral capsid protein VP5 in the treated Huh7 xenograft tumors. Black arrows indicate foci of viral replication. The scale bar in (**C**) represents 2000 μm

The angiogenesis of the tumors was quantified. The number of terminal branches of the vessels was calculated in the native tumors before weighing. The H2252 treated tumors had a 60% reduction in vessel branching compared to PBS treatment (*p* = 0.005), whereas H2254 caused a reduction of only 13% (*p* = 1) (Fig. 1B). The vector treated tumors did not differ significantly in either weight or area from the control (PBS) treated tumors (Fig. 2A).

Half of the tumor was homogenized and viral titers quantified with plaque titration. Tumors treated with both H2252 and H2254 had recoverable titers of about 10^4^ pfu per tumor (Fig. 2B). q-RT-PCR revealed DCN mRNA expression in H2252 treated tumor halves, with copy numbers ranging from 267 431 to 5 529 356 per tumor, while control tumors had no detectable expression. The other half of the tumor was histologically examined, and viral foci of replication on the peripheries of the tumors were detected (Fig. 2C).

## Discussion

Liver cancer is a major cause of cancer related deaths despite modern treatments. We have previously constructed a DCN-expressing herpes simplex vector to combine virus-mediated oncolysis and the oncosuppressive and anti-angiogenic effects of DCN. A suitable model to study the effect of the novel gene therapy on angiogenesis is the CAM model, with Huh7 cell xenografts representing liver cancer. To our knowledge, this is the first study showing the effect of herpes simplex virus-mediated DCN gene therapy against tumor angiogenesis. This is also one of the first studies documenting the utilization of CAM models for evaluating oncolytic HSV vectors.

Immunoblotting confirmed that Huh7 cells infected with H2252 produce DCN, whereas no native expression of DCN in these cells could be detected (Fig. 1A). This corresponds with previous findings of hepatic cancer being devoid of DCN expression [5]. We could not see any oncolysis in Huh7 cells when infected with HSV vectors (results not shown), owing to Huh7 cells’ retained ability for interferon signaling [11]. In this regard, DCN’s effect on the tumor is more apparent as the virus does not interfere with the results.

Huh7 cell tumors in chicken embryos were treated with 10^7^ pfu of H2252 or H2254. The chicken embryos showed no sign of infection or disease caused by the HSV vector. H2252 treatment reduced the vessel branching by 60% (*P* = 0.005) (Fig. 1B). The treatment did not affect the weights or areas of the tumors (Fig. 2A). We could also recover replicative virus from the tumors, and histological sampling revealed viral replication on the peripheries of the tumors as an indicator of functional gene therapy (Fig. 2B, C**)**. As the primary antibody for detecting the vector in the sample was for the major capsid protein VP5, only intracellular replicative viruses were detected. It is unclear how this incomplete penetration affects the results, however, DCN is a secreted protein and will most likely have a broader effect on the tumor than the limit of the infection. The localization also resembles HSV vector tumor penetration in clinical trials [15]. We find that these results are promising, as a single-dose treatment was given on already vascularized tumors 4 days prior to collection.

These results show that the CAM model is a suitable method to study the anti-angiogenic effects of HSV-mediated gene therapies with an overlay administration method. The Huh7 cells were suitable for studying the effect of therapeutic transgenes without the interference of oncolysis caused by the HSV vector. This study’s biggest limitations include non-standardized sizes of the tumors, inability to follow tumor growth, the short time frame of the treatment, the incomplete penetration of the vector into the tumor, as well as a lack of studies on angiogenic factors. To overcome these limitations, further studies in another tumor model such as mice is required. However, this study demonstrates that DCN expression by HSV reduces tumor angiogenesis and justifies the continuation of the development of a DCN-expressing HSV vector for the treatment of cancer.

## Data Availability

Data and materials are available upon request.
